# Effects of resistance exercise intensity on cerebral blood flow and cerebrovascular reactivity in healthy young males: A pilot study

**DOI:** 10.14814/phy2.70361

**Published:** 2025-05-12

**Authors:** Zusheng Li, Haibin Liu, Mengzhen Li, Shuhan Liu, Xin Pan, Hongling Zhao, Chundong Xue, Dong Xu

**Affiliations:** ^1^ Department of Neurology Central Hospital of Dalian University of Technology Dalian Liaoning China; ^2^ School of Sport and Health Sciences Dalian University of Technology Dalian Liaoning China; ^3^ School of Biomedical Engineering, Faculty of Medicine Dalian University of Technology Dalian Liaoning China

**Keywords:** blood pressure, cerebral hemodynamics, cerebrovascular reactivity, exercise intensity, resistance exercise

## Abstract

Exercise intensity has been shown to elicit different cerebral blood flow velocity (CBFv) responses. This study aimed to compare the acute effects of resistance exercise at different intensities on hemodynamics and cerebrovascular reactivity in healthy young males. Eleven healthy young males were recruited, and three trials of dumbbell alternating curls were performed in order of increasing intensity: low (30%–35% 1RM), medium (55%–60% 1RM), and high (75%–80% 1RM). Blood pressure, heart rate, CBFv, and cerebrovascular reactivity were measured at baseline, 1, 5, and 10 min after exercise. (1) At 10 min, the mean blood pressure in the medium‐intensity was significantly lower than that at baseline. (2) At 1 min, the systolic velocity of the middle cerebral artery in the medium‐intensity increased significantly. (3) At 1 min, the pulsatility index (PI) and resistance index (RI) of the high‐intensity increased significantly. At 10 min, the PI and RI of the medium‐intensity were significantly lower than the baseline. (4) At 5 min, the breath‐holding index significantly increased in the medium‐intensity but decreased in the high‐intensity. Compared with low and high intensities, medium‐intensity resistance exercise may be more effective in lowering blood pressure and enhancing cerebral hemodynamics.

## 1 INTRODUCTION

Adequate and continuous blood supply is essential to maintain the best brain function. Cerebral blood flow (CBF) decreased with age (Stoquart‐ElSankari et al., 2007). Aging‐induced cerebral artery endothelial dysfunction is a major risk factor for cerebrovascular disease (Karlsson et al., 2017), such as stroke (Markus et al., 2004), Alzheimer's disease (Kisler et al., 2017), and cognitive decline (Chrissobolis et al., 2011). Regular exercise may be a useful non‐pharmacological intervention to improve cerebral blood flow and regulate cerebral hemodynamics, including pulsatility index, resistance index, and cerebrovascular reactivity (Bailey et al., 2013; Zimmerman et al., 2014).

At present, there are many studies on the effect of exercise on cerebral blood flow, but most of these studies are based on aerobic exercise. A consensus has been reached on the response of cerebral hemodynamics to aerobic exercise (Braz & Fisher, 2016; Weston et al., 2021; Witte et al., 2019). With the increase of exercise intensity, the response of cerebral blood flow is an inverted U‐pattern, and the peak of CBF appears at 50%–80% of the maximum aerobic capacity (VO_2_max); at maximum exercise, hypocapnia‐induced CBF decreased significantly (Smith & Ainslie, 2017). However, there are few studies on the influence of resistance exercise on cerebral hemodynamics, and most of the exercise interventions used are hand grip (Braz et al., 2014; Fernandes et al., 2016) or lower limb movements such as upright squats (Perry, Schlader, et al., 2014), leg presses (Moralez et al., 2012) and leg stretches (Hirasawa et al., 2016).

Previous studies have confirmed and recommended resistance exercise to improve muscle mass and *reduce* cerebral blood flow decline caused by aging (Landi et al., 2014). In addition, brain‐derived neurotrophic factor temporarily increased immediately after RE (Marston et al., 2017; Yarrow et al., 2010), which is a key biomarker related to neurogenesis and neuron survival (Mattson et al., 2004). Furthermore, RE is recommended as a treatment for clinical cohorts, including stroke (Billinger et al., 2014) and neurodegenerative diseases (Larson et al., 2006).

A recent review shows cerebrovascular responses are inconsistent in different resistance exercise patterns (Perry & Lucas, 2021). The reason for this difference may be that the number, speed, range, and quality of the actions completed by the participants are different. At present, there is only one study that focuses on the effect of upright squatting on cerebral blood flow under different exercise intensities (Perry, Schlader, et al., 2014), but more cerebral hemodynamic parameters were not measured, including pulsatility index, resistance index, and cerebrovascular reactivity. The vasodilation response of intracranial arteries to the increase of PCO_2_ is called cerebrovascular reactivity (CVR) (Howe et al., 2020). Cerebrovascular reactivity can be used as an important biomarker to predict neurodegenerative diseases (Ryman et al., 2023). The acute effect of exercise on CVR is not clear, especially the response of CVR to resistance exercise at different intensities. A study has shown that individuals after resistance exercise show higher central arterial stiffness and CBF pulsatility, which can lead to brain microvascular damage, impair buffer function, and increase the risk of cerebrovascular disease (Nakamura & Muraoka, 2018). However, there is no research on the acute effect of different exercise intensities on CBF pulsatility. The chronic adaptation of vascular function to long‐term exercise comes from the accumulation of transient changes in hemodynamics after a single acute exercise (Dawson et al., 2018). Therefore, it is important to study the acute changes in cerebral hemodynamics after a single exercise.

Up to now, there has been no study on the effect of dynamic resistance exercise of upper limbs (non‐hand grip) with different intensities on cerebral hemodynamics. Therefore, the purpose of this study is to explore the acute effects of dumbbell curl exercise with different exercise intensities on cerebral hemodynamic parameters and cerebrovascular reactivity, so as to choose *a* safer and more beneficial exercise intensity, improve CBF and cerebrovascular reactivity, and promote the chronic reconstruction of cerebrovascular function.

## 2 MATERIALS AND METHODS

### 2.1 Participants

Eleven healthy young male undergraduates (mean age: 19.8 ± 2.2 years) were recruited (Table 1). None of the participants had professional resistance training experience. Exclusion criteria included a history of cardiovascular, cerebrovascular, metabolic, or respiratory disorders; Musculoskeletal injuries; Smoking. All participants were asked to avoid caffeine and alcohol 24 h before the experiment and to avoid any strenuous exercise and resistance exercise within 72 h before each experiment. Considering the effect of circadian rhythms on cerebral blood flow, all experiments were carried out in the afternoon. This study complied with the Declaration of Helsinki and was approved by the Ethics Committee of Dalian University of Technology. The participants provided their written informed consent to participate in this study.

**TABLE 1:** Baseline characteristics of participants (*n* = 11).

	Value (mean ± SD)
Age (years)	19.80 ± 2.20
Height (cm)	181.06 ± 5.54
Weight (kg)	71.02 ± 6.06
Body mass index (kg/m^2^)	21.67 ± 1.75
Body fat percentage	16.21 ± 3.99

### 2.2 Experimental design

Height, weight, BMI, body fat percentage, and blood pressure were measured when the participants came to the laboratory for the first time. Participants then performed a 10‐min upper‐limb warm‐up, including static and dynamic stretches of the shoulder and wrist joints. One‐Repetition Maximum (1RM) was measured by increasing the load by 0.5 kg until the participant could not contract the biceps brachii to the standard motion. Based on the 1RM of dumbbell curls, 30%–35% 1RM was defined as low‐intensity, 55%–60%1RM as medium‐intensity, and 75%–80%1RM as high‐intensity. The formal experiment was conducted at least a week after the first visit. The low‐intensity trial in the progressive loading protocol was used as a warm‐up for the high‐intensity trial, which reduces the risk of sports injury during the high‐intensity trial, and the progressive load is more in line with the 1RM measured in the previous period. At the same time, considering that human cerebral blood flow will be affected by emotions, sleep, circadian rhythms, etc., in order to avoid baseline differences, we decided to conduct experiments on the same day in the order of increasing intensity. On the day of the formal experiment, all participants need to complete three trials of upper limb dumbbell alternating curling exercises with different intensities in the order of increasing intensity. The interval between each two trials is at least 30 min. To avoid the impact of potential circadian rhythm changes on hemodynamics, each participant was tested at the same time in the afternoon. Three intensity trials each need to complete three sets of alternating dumbbell curls, left hand and right hand alternately, each completed 8 times. Each set was separated by a 2‐min interval.

**FIGURE 1:**
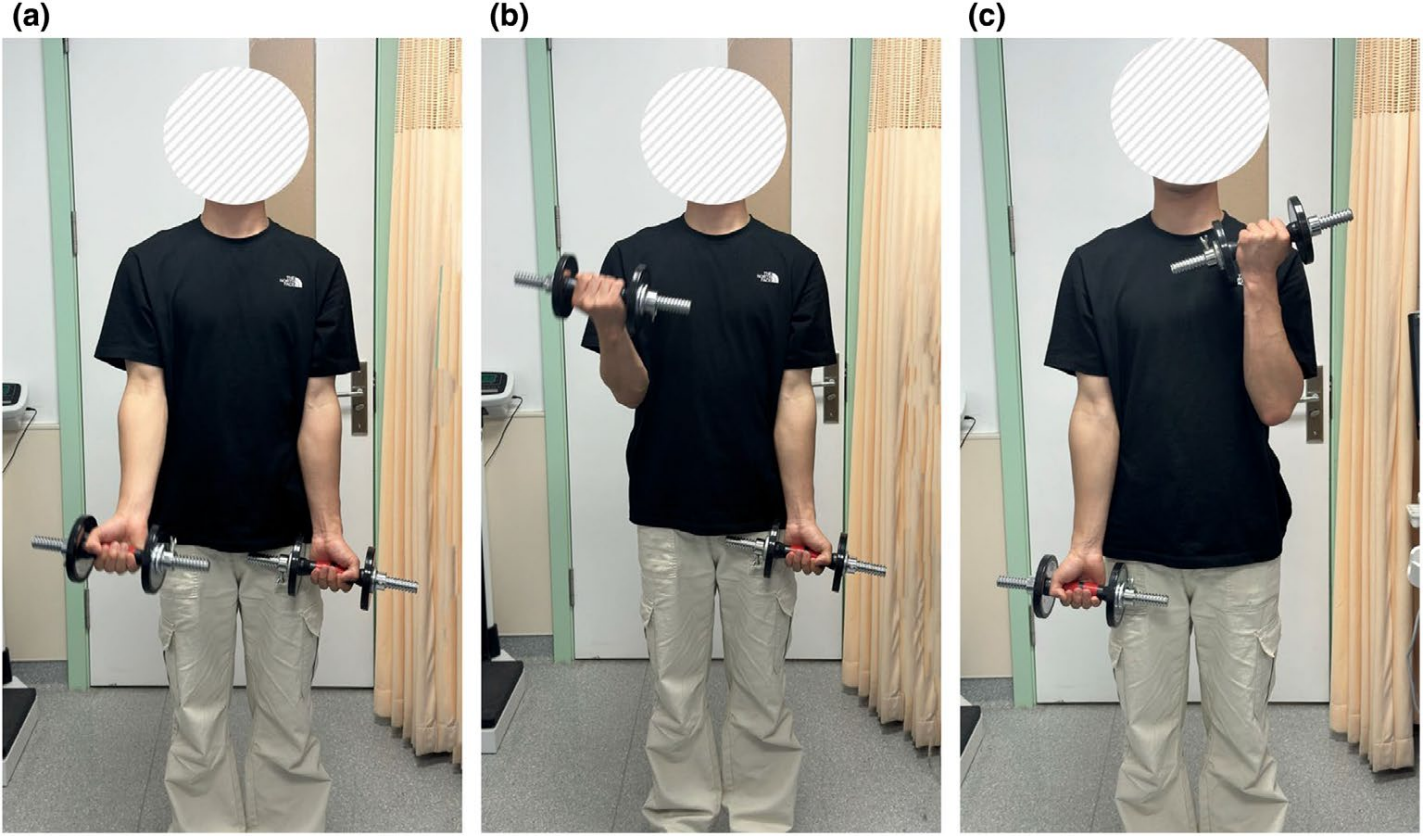
Movement sequence during the resistance exercise intervention. (a) Starting static posture. (b) Right‐arm dumbbell curl at peak contraction. (c) Left‐arm dumbbell curl at peak contraction. The participant in Figure 1 consented to the publication of the image in an anonymized form with the face masked.

In this study, the details of dumbbell alternating curls are shown in (Figure 1). (1) When standing, the upper arm is clamped on both sides of the chest. (2) During each repetition, wrist rotation is restricted to maintain consistent joint positioning. (3) In the initial position, the palm is facing forward. (4) When curling, it contracts centripetally to the maximum angle, and then extends back down toward the lateral side of the thigh. The movement rhythm is controlled by the metronome (1 s concentric and 1 s eccentric). (5) The participants were asked to match the breathing rhythm with the movement rhythm (exhale during dumbbell lifting and inhale during falling), and to inform and supervise the participants to maintain a stable degree of breathing, to prevent breath holding and hyperventilation. However, slight Valsalva Maneuver (VM) is inevitable in the last few contractions of the high‐intensity trial.

**FIGURE 2:**
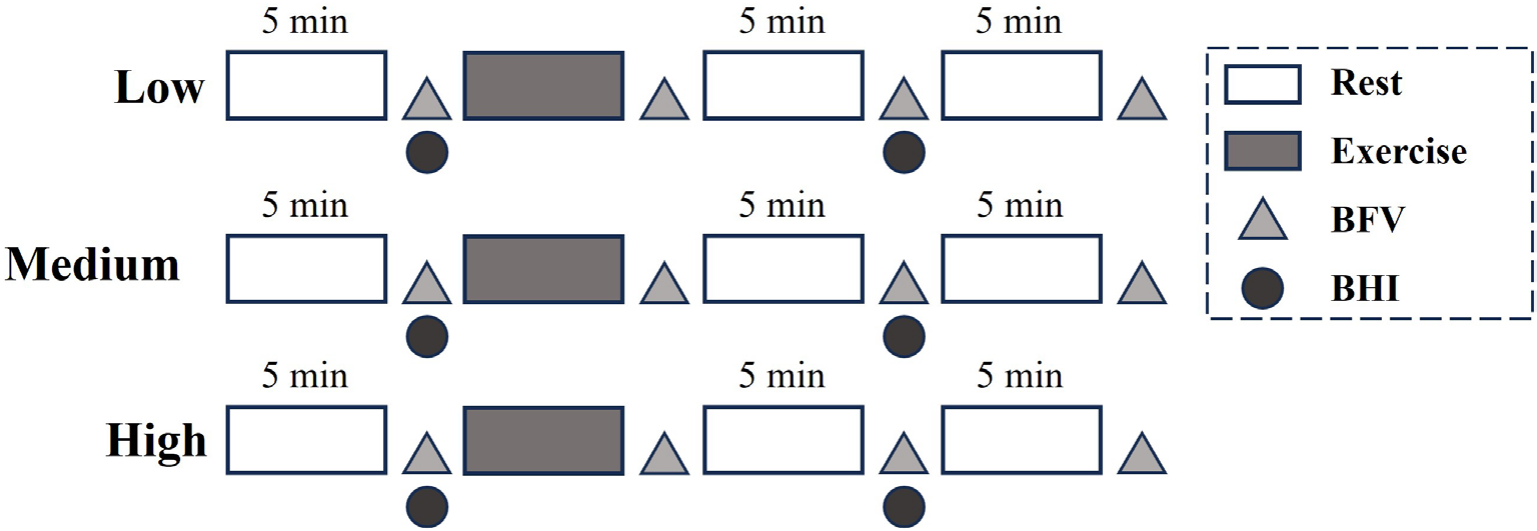
Resistance exercise intervention protocol. Participants completed three trials at low, medium, and high intensities. Measurements were taken at baseline (BL), 1, 5, and 10 min post‐exercise. BFV, Blood flow velocity; BHI, Breath‐holding index.

The experimental process is shown in (Figure 2). In the formal experiment, participants' baseline (BL) parameters were measured before each trial, including left middle cerebral artery hemodynamics, cerebrovascular reactivity, brachial blood pressure, and heart rate. After 5 minutes of warm‐up (static stretches and dynamic stretches), the trial was carried out, and the hemodynamics of left middle cerebral arteries, brachial blood pressure, and heart rate were measured repeatedly at the time points of 1, 5, and 10 min after exercise. Cerebrovascular reactivity was measured at 5 min after exercise. After resting quietly for at least 30 minutes, the trial was conducted in the order of increasing intensity, and the data collection scheme was the same as above.

### 2.3 Measurements

#### 2.3.1 Blood pressure and heart rate

Participants were placed in a supine position. Heart rate, systolic, and diastolic blood pressure were measured on the left arm using a cuff‐type electronic manometer (HEM‐7136, Omron, Japan).

#### 2.3.2 Cerebral blood flow velocity

The velocity of the participants' left middle cerebral artery was measured by using transcranial Doppler (TCD) (EMS‐9D PRO) before and after each trial. The participants were in the supine position; the maximum systolic velocity (MCAvs), minimum diastolic velocity (MCAvd) and mean velocity (MCAvm) of the middle cerebral artery were collected from the temporal window using a 2 MHz TCD probe at a depth of 50–60 mm. The middle cerebral artery was located using a recognized standard, including the position and direction of the probe and the depth of the ultrasound (Willie et al., 2011). The cerebral hemodynamic parameters of each participant were measured by a professional doctor at the same detection depth and position. After seven complete and clear cardiac cycle signals were identified by TCD, the average values of each index were calculated. Cerebral hemodynamic parameters PI and RI were used as indexes to measure cerebral arterial resistance and were calculated by the following formulas:

$$\mathrm{PI}=\left(\text{MCAvs}-\text{MCAvd}\right)/\text{MCAvm}$$

$$\mathrm{RI}=\left(\text{MCAvs}-\text{MCAvd}\right)/\text{MCAvs}.$$

#### 2.3.3 Cerebrovascular reactivity

The breath‐holding index (BHI) is a reliable indicator for evaluating cerebrovascular reserve impairment, with reductions in BHI associated with cognitive dysfunction (Bian et al., 2019). First, the participants were in the supine position, then the mean cerebral blood flow velocity (MCAvmean) at rest was measured and recorded. Then, participants were asked to hold their breath for 30 s after a normal inspiratory breath to exclude the Valsalva Maneuver. Participants who could not hold their breath for 30 s were asked to hold their breath for as long as possible, and this time was used for subsequent calculations. The maximum of MCAvm in the process of breath‐holding measurement is recorded as MCAvmax. BHI was calculated by (MCAvmax − MCAvmean)/(MCAvmean × breath holding time) × 100.

### 2.4 Statistical analysis

All data were presented as the mean ± SD. Two‐way repeated measures analysis of variance (ANOVA) was used to examine the effects of different exercise intensities on middle cerebral artery velocity and hemodynamic variables across four assessment time points (BL and 1, 5, and 10 min). The level of statistical significance was set at *p* < 0.05. Effect sizes (Cohen's d) were calculated to quantify the standardized difference between means.

## 3 RESULTS

### 3.1 Effects of different intensity resistance exercise on blood pressure and heart rate

**FIGURE 3:**
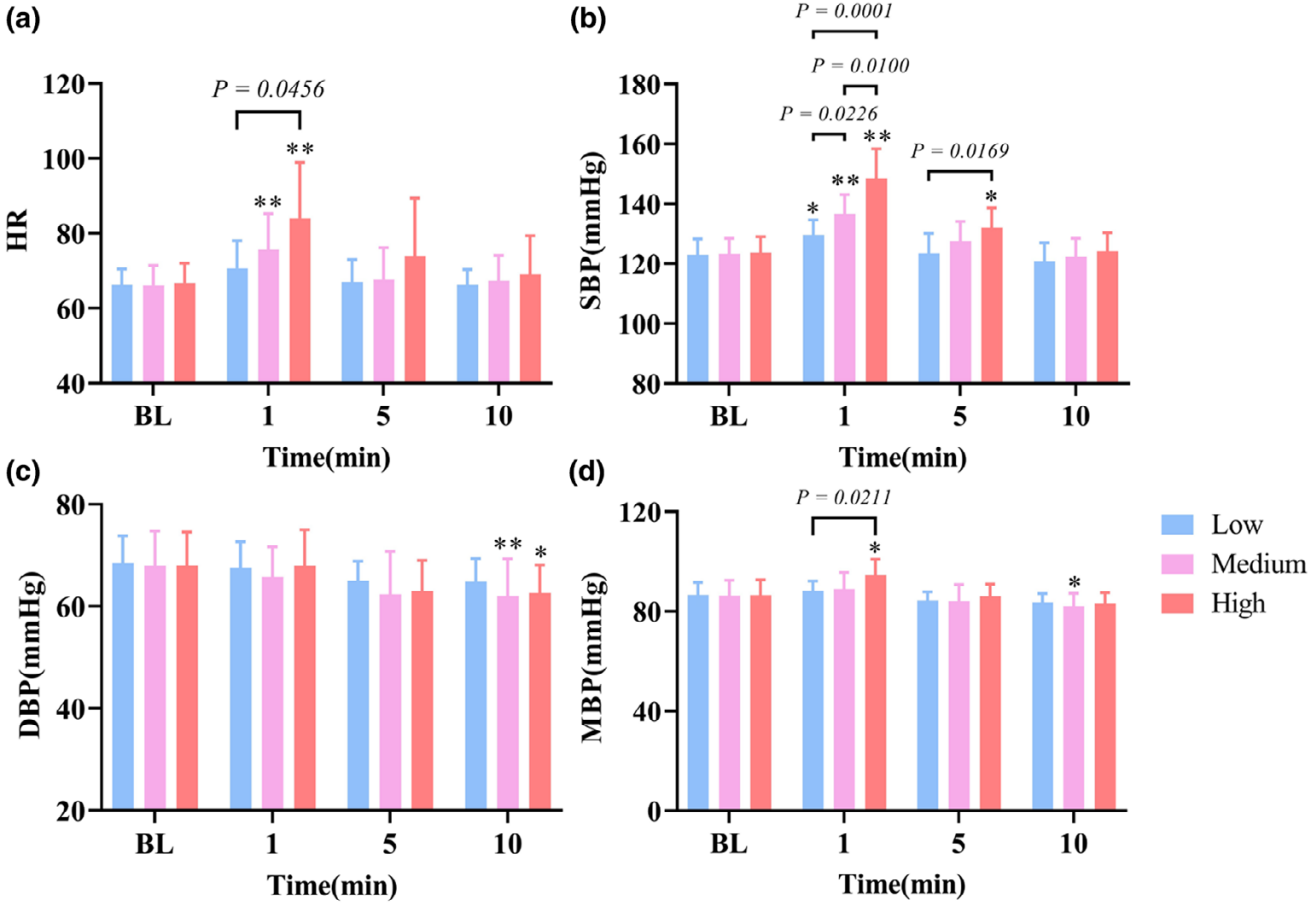
Effects of different resistance exercise intensities on blood pressure and heart rate. (a) Heart rate (HR). (b) Systolic blood pressure (SBP). (c) Diastolic blood pressure (DBP). (d) Mean blood pressure (MBP). *p < 0.05 versus BL. **p < 0.01 versus BL.

As shown in Figure 3, at 1 min, HR increased significantly in medium‐intensity (*p* = 0.007, *d* = 1.237) and further escalated at high‐intensity (*p* = 0.0052, *d* = 1.533). There was a significant difference between low‐intensity and high‐intensity (*p =* 0.0456, *d* = 1.132). At 1 min, the systolic blood pressure (SBP) of three trials increased significantly (*p* < 0.05), and the increase was positively correlated with the intensity. The mean blood pressure (MBP) of high‐intensity (*p* = 0.0012, *d* = 1.346) was increased significantly at 1 min, which was significantly different from that of the low‐intensity trial (*p* = 0.0211, *d* = 0.93). At 10 min, the diastolic blood pressure (DBP) of the medium‐intensity (*p* = 0.0064, *d* = 0.833) and high‐intensity (*p* = 0.0293, *d* = 0.89) was significantly lower than that of BL, and the MBP of the medium‐intensity decreased significantly (*p* = 0.0208, *d* = 0.784).

### 3.2 Effects of different intensity resistance exercise on cerebral blood flow and hemodynamics

**FIGURE 4:**
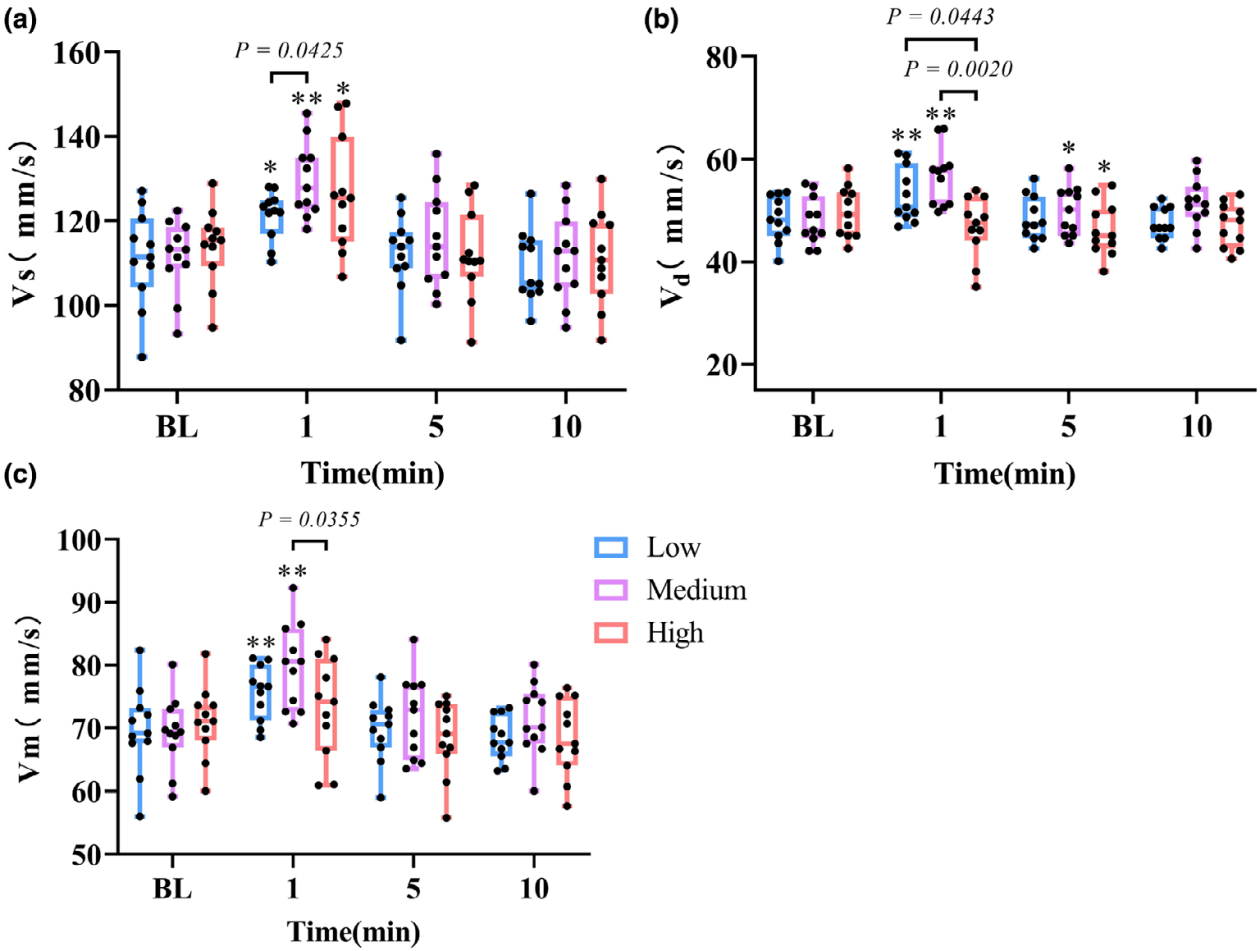
Acute effects of resistance exercise intensities on middle cerebral artery velocities: (a) systolic (MCAvs), (b) diastolic (MCAvd), and (c) mean velocity (MCAvm). Boxes represent the interquartile range (25th to 75th percentile), with horizontal lines indicating the median. Whiskers represent minimum and maximum values. *p < 0.05 versus BL. **p < 0.01 versus BL.

**FIGURE 5:**
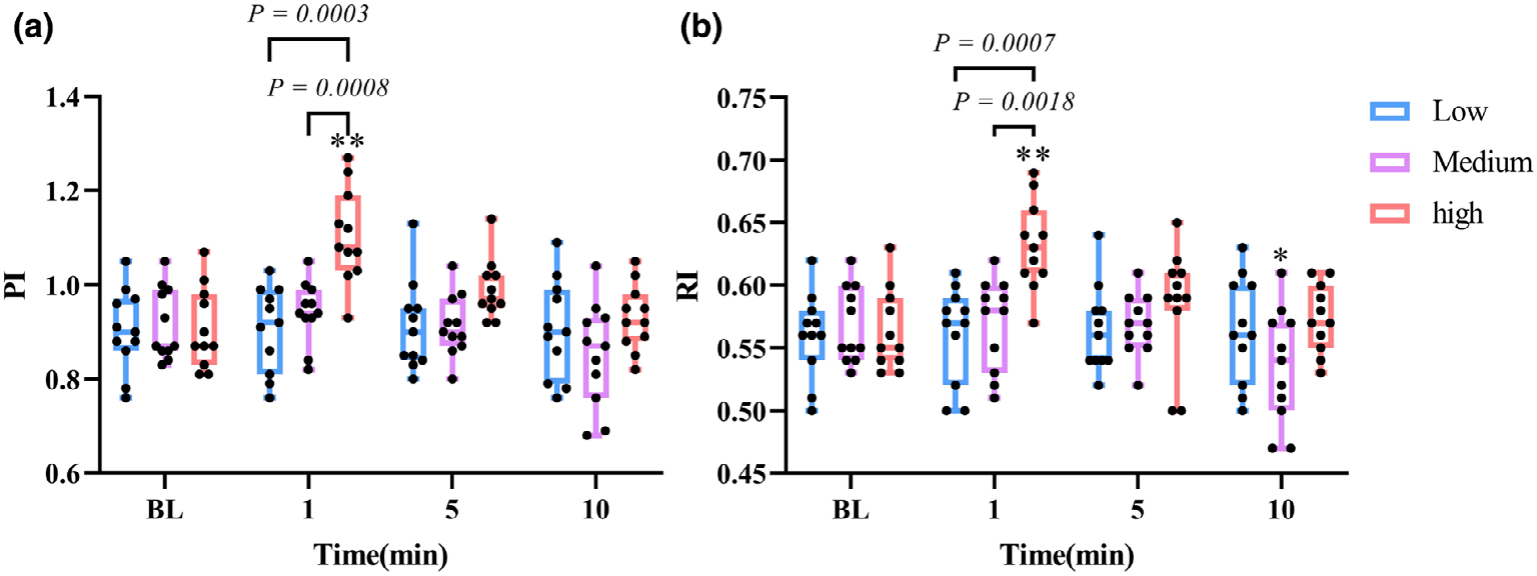
Acute effects of different resistance exercise intensities on (a) pulsatility index (PI) and (b) resistance index (RI). Boxes represent the interquartile range (25th to 75th percentile), with horizontal lines indicating the median. Whiskers represent minimum and maximum values. *p < 0.05 versus BL. **p < 0.01 versus BL.

As shown in Figure 4, at 1 min, the maximum systolic velocity of the middle cerebral artery (MCAvs) of three trials increased significantly (*p* < 0.05), especially in the medium‐intensity trial (*p* < 0.0001, *d* = 1.834). There is a significant difference between medium and low‐ intensity (*p* = 0.0425 *d* = 1.173). The minimum diastolic velocity of the middle cerebral artery (MCAvd) of low (*p* = 0.0204, *d* = 0.941) and medium‐ intensity (*p* = 0.0011, *d* = 1.582) immediately significantly increased at 1 min. However, the MCAvd of high‐intensity trials showed a downward trend, which was significantly different from that of low‐ intensity (*p* = 0.0443, *d* = 1.106) and medium‐ intensity trials (*p* = 0.0020, *d* = 1.714). At 5 min, the MCAvd of the medium‐intensity trial was still significantly higher than that of the BL (*p* = 0.045, *d* = 0.635), but the MCAvd of the high‐intensity trial was significantly lower than that of the BL (*p* = 0.0339, *d* = 0.774). The mean velocity of the middle cerebral artery (MCAvm) of low‐ intensity (*p* = 0.0072, *d* = 1.028) and medium‐ intensity trials (*p* = 0.0009, *d* = 1.675) increased significantly at 1 min, and reached the peak in the medium‐intensity trial. The MCAvm in the high‐intensity trial showed the smallest increase. The MCAvm of medium‐ intensity was significantly higher than that of high‐ intensity (*p* = 0.0355, *d* = 0.915) at 1 min. It can be observed in Figure 5 that the pulsatility index (PI) and resistance index (RI) of medium and low‐ intensity trials did not change significantly, while the PI (*p* = 0.0002, *d* = 1.797) and RI (*p* = 0.0004, *d* = 1.854) of the high‐intensity trial increased significantly, which was significantly different from that of the low and medium‐ intensity trials (*p* < 0.001). At 10 min, the RI in the medium‐intensity trial was significantly lower than that at BL (*p* = 0.0392, *d* = 0.774).

### 3.3 Effects of different intensity resistance exercise on cerebrovascular reactivity

**FIGURE 6:**
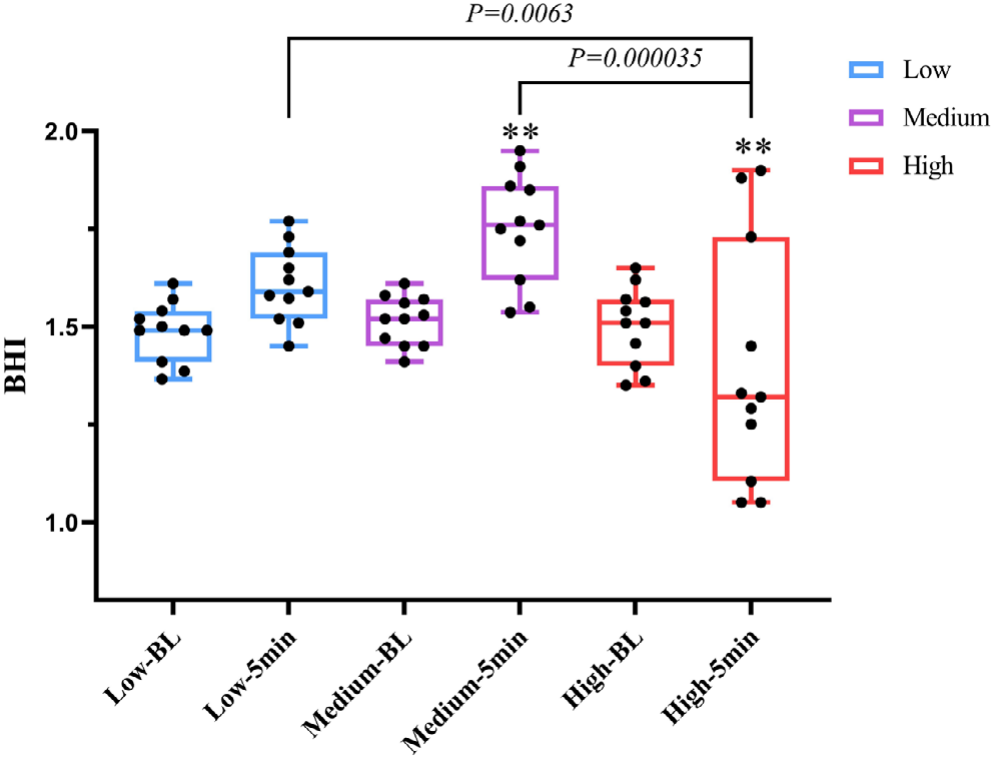
Acute effects of different resistance exercise intensities on the breath‐holding index (BHI). Boxes represent the interquartile range (25th to 75th percentile), with horizontal lines indicating the median. Whiskers represent minimum and maximum values. *p < 0.05 versus BL. **p < 0.01 versus BL.

Figure 6 reflects the change and distribution of cerebrovascular reactivity after exercise in different intensity trials. At 5 min after exercise, the BHI of low‐intensity (*p =* 0.1479, *d* = 1.325) and medium‐intensity (*p =* 0.0009, *d* = 2.013) trials showed an upward trend. However, the BHI of the high‐intensity trial decreased significantly (*p =* 0.0025, *d* = 0.589), which was significantly different from that of low (*p =* 0.0063, *d* = 0.786) and medium‐intensity (*p =* 0.000035, *d* = 1.457).

The results of the two‐way repeated measures ANOVA for all measured indicators are summarized in Table 2, showing the main effects of exercise intensity, time, and their interaction effect. Most variables demonstrated significant main effects of both intensity and time. Significant interaction effects were observed in SBP, Vd, and PI.

**TABLE 2:** Summary of two‐way repeated measures ANOVA results for all indicators.

Indicator	Intensity effect (*p*‐value)	Time effect (*p*‐value)	Interaction effect (*p*‐value)
HR	0.0031	<0.0001	0.0614
SBP	<0.0001	<0.0001	<0.0001
DBP	0.0613	<0.0001	0.9302
MBP	0.0166	<0.0001	0.1175
Vs	0.2461	<0.0001	0.6465
Vd	<0.0001	0.0021	0.0007
Vm	0.0160	<0.0001	0.1549
PI	<0.0001	0.0003	0.0034
RI	0.0002	0.0004	0.1548
BHI	0.1914	<0.0001	0.4962

## 4 DISCUSSION

The stimulation of single acute exercise on hemodynamics will have a direct impact on the vascular system. Long‐term regular exercise will accumulate acute effects and lead to chronic remodeling of vascular structure and function (Green et al., 2017). This study mainly compares the effects of different exercise intensities on cerebral hemodynamics and cerebrovascular reactivity. The main findings are as follows: Medium‐intensity dumbbell curls resistance exercise has more positive and acute effects on cerebral hemodynamics and cerebrovascular reactivity.

### 4.1 Response of blood pressure and heart rate to different exercise intensities

A previous study found that 70% of 1RM leg press exercises immediately obtained the peak values of HR, DBP, and SBP (de Sousa et al., 2014). Our research shows that the increase in SBP immediately after exercise is linearly related to the intensity of exercise. The increase in blood pressure is attributed to greater vascular resistance and the amount of cardiac output reached at the end of exercise. The increase in intramuscular pressure caused by the increase in exercise intensity will limit blood flow to muscle, and with the accumulation of metabolites in muscle, it will trigger metabolic reflexes and activate the sympathetic nervous system (de Sousa et al., 2014). Unlike aerobic exercise, the Valsalva Maneuver (VM), a key factor in maintaining trunk stability during resistance training, can acutely elevate intrathoracic pressure, thereby impairing venous return and destabilizing cerebral perfusion. When the exercise intensity reaches a high load, the inevitable VM will gradually increase the intrathoracic pressure, leading to an acute increase in blood pressure (Perry & Lucas, 2021). Studies have shown that increased expiratory muscle activity leads to increased sympathetic vasomotor outflow and blood pressure (Katayama et al., 2015). It has been recommended to engage in resistance exercise to lower blood pressure (Casonatto et al., 2016). Our results show that the DBP and MBP in the medium‐intensity trial decreased most significantly 10 min after exercise. This means that compared with high‐intensity exercise, medium‐intensity exercise reduces the negative effects caused by sympathetic overexcitation and has a lower rating of perceived exertion and lower blood lactate (Pinto et al., 2018). Therefore, long‐term medium‐intensity resistance exercise is more effective in reducing blood pressure.

### 4.2 Response of hemodynamics to different exercise intensities

Our results show that with the increase in intensity, MCAvm has an inverted U‐pattern. We found that the main factor for the decrease in MCAvm in the high‐intensity exercise trial occurred via a load‐dependent decrease in MCAvd, which would lead to more severe blood flow fluctuation; this was also the reason for the significant increase in PI and RI under high‐intensity resistant exercise. The results of (Perry, Schlader, et al., 2014) show that MCAvm remained below the baseline for the longest time after the 90% six‐repetition maximum load, and it takes the longest time to recover. Previous studies have found that compared with sedentary people, the MCAvm of endurance exercise individuals at rest increases by about 17% for life (Ainslie et al., 2008). A previous study found that individuals who often performed resistance exercise showed higher central arterial stiffness and PI, and lower arterial compliance, suggesting decreased vascular buffering function (Nakamura & Muraoka, 2018). Compared with rest, acute and chronic high‐intensity intermittent exercise will reduce the dCA phase and MCAv (Whitaker et al., 2020). These studies are similar to the trend reflected by our results. However, the response of cerebral blood flow to different exercise intensities is not always similar. One study compared the effects of running and cycling on cerebral blood flow under different exercise intensities and found that the MCAv of cycling is an inverted U‐pattern, while the MCAv of running fluctuates (Furlong et al., 2020). In contrast, the PI of the running trial is an inverted U‐pattern, while the PI of cycling increases linearly. This shows that the effects of cycling on PI are similar to those of resistance exercise, which may be due to the trigger of VM during high‐intensity resistance exercise. In this study, not all participants' cerebral blood flow decreased in the high‐intensity trial, and a few participants with prior resistance training experience may exhibit better control of the VM, so their cerebral blood flow further increased, but VM will inevitably occur when the intensity is too high. The high‐intensity trial in this study included only three sets of dumbbell curls; thus, a more pronounced trend may emerge with continued increases in load. This means that the appearance of VM may be a sign of the change in cerebral blood flow response mode during different intensity exercises. The VM is generally avoidable at low intensity but becomes nearly inevitable during high‐intensity resistance exercises. (Perry, Cotter, et al., 2014) found that compared with 30% efforts, 90% of the maximum 1 RM resulted in a significant decrease in cerebral blood flow velocity and oxygenation. This is also a common cause of syncope after high‐intensity resistance exercise.

### 4.3 Response of cerebrovascular reactivity to different exercise intensities

At present, there is no systematic summary of the effects of different exercise patterns and intensities on cerebrovascular reactivity. (Murrell et al., 2013) found that cerebrovascular reactivity increased after 12 weeks of low‐intensity aerobic exercise training intervention, whether at rest or during acute exercise. (Ogoh et al., 2008; Rasmussen et al., 2006) found that hypercapnia reactivity increased by about 30% induced by 67% VO_2_max. However, high‐intensity interval exercise HIIE acutely decreased cerebrovascular reactivity to higher CO_2_ compared to rest and medium intensity (Whitaker et al., 2020). These studies suggest that cerebrovascular reactivity also follows an inverted U‐shaped response to exercise intensity, consistent with our findings, despite their use of aerobic rather than resistance exercise interventions. It is reported that cerebrovascular reactivity is associated with maximal aerobic capacity in healthy older adults (Barnes et al., 2013), so we speculate that the cerebrovascular reactivity of a few participants did not decrease under high‐intensity resistance may be related to better cardiopulmonary health. Cerebrovascular reactivity can serve as an important biomarker and target for future interventions (Ryman et al., 2023), and it has been proved that age and gender have a significant effect on cerebrovascular reactivity, so our experimental participants are all male and of similar age (Chen et al., 2024). Healthy people usually have better vascular function and higher vascular compliance. Low to medium‐intensity exercise enhances the balance of sympathetic and parasympathetic nerves, promotes the release of vasodilatory factors (such as nitric oxide), improves cerebrovascular reactivity, and leads to an increase in BHI. The superior effect of medium‐intensity resistance exercise on cerebral hemodynamics may be related to the bioavailability of nitric oxide (NO). NO is a gas transmitter and regulator of numerous biochemical processes. With the increase of age, the decrease of NO bioavailability was especially obvious in sedentary individuals, while individuals who were often engaged in physical activity maintained a high level of NO (Shannon et al., 2022). High‐intensity exercise can significantly activate the sympathetic nervous system, resulting in a sharp increase in blood pressure and heart rate, causing cerebral vasoconstriction and increased arterial stiffness (Nobrega et al., 2014). In addition, due to short‐term peripheral vasoconstriction to maintain blood pressure stability, the sensitivity of cerebral blood vessels to carbon dioxide may be temporarily reduced, weakening the ability to respond to carbon dioxide, resulting in a decrease in BHI.

## 5 CONCLUSIONS

In a trial of healthy young male individuals, we showed that medium‐intensity resistance exercise elicited more favorable acute changes in cerebral hemodynamics and cerebrovascular reactivity compared to low and high intensity. High‐intensity resistance exercise may have a negative effect on cerebrovascular reactivity. Although other cerebral and peripheral vascular responses were not assessed concurrently, medium‐intensity resistance training may serve as an effective strategy to enhance cerebral perfusion, but its clinical significance needs to be verified, especially in people with potentially impaired cerebrovascular regulation. In addition, all participants were healthy young males, which may limit the generalizability of the results to other populations.

## FUNDING INFORMATION

This research was supported by the National Natural Science Foundation of China (Grant No. 12172081), the Fundamental Research Foundation for the Central Universities in China (DUT22YG216), and the Life and Health Guidance Program Project (2022ZXYG03).

## CONFLICT OF INTEREST STATEMENT

## ETHICS STATEMENT

The study protocol was approved by the Ethics Committee of Dalian University of Technology and complied with the Declaration of Helsinki. All participants provided written informed consent. The participant in Figure 1 consented to publication of the image in an anonymised form with the face masked.

## Data Availability

The datasets generated and analyzed during the current study are available from the corresponding author upon reasonable request.
